# Discovery on Antibiotic Resistance Patterns of *Vibrio parahaemolyticus* in Selangor Reveals Carbapenemase Producing *Vibrio parahaemolyticus* in Marine and Freshwater Fish

**DOI:** 10.3389/fmicb.2018.02513

**Published:** 2018-10-25

**Authors:** Learn-Han Lee, Nurul-Syakima Ab Mutalib, Jodi Woan-Fei Law, Sunny Hei Wong, Vengadesh Letchumanan

**Affiliations:** ^1^Novel Bacteria and Drug Discovery Research Group, Biomedicine Research Advancement Centre, School of Pharmacy, Monash University Malaysia, Bandar Sunway, Malaysia; ^2^Biomedical Research Laboratory, Jeffrey Cheah School of Medicine and Health Sciences, Monash University Malaysia, Bandar Sunway, Malaysia; ^3^Center of Health Outcomes Research and Therapeutic Safety, School of Pharmaceutical Sciences, University of Phayao, Phayao, Thailand; ^4^UKM Medical Molecular Biology Institute, UKM Medical Centre, National University of Malaysia, Bangi, Malaysia; ^5^Department of Medicine and Therapeutics, Li Ka Shing Institute of Health Sciences, The Chinese University of Hong Kong, Shatin, Hong Kong

**Keywords:** *Vibrio parahaemolyticus*, carbapenem, freshwater, marine, antibiotic resistant, MAR index

## Abstract

*Vibrio parahaemolyticus*, a Gram-negative halophilic bacterium is often associated with fish and fishery products, thus causing gastroenteritis in humans upon ingestion of contaminated food. *V. parahaemolyticus* has become a globally well-known pathogen with yearly reported cases in many countries. This study aimed to discover the antibiotic resistance patterns of *V. parahaemolyticus* as well as detect Carbapenem resistant isolates from marine and freshwater fish in Selangor. A total of 240 freshwater and marine fish samples collected from wet market and supermarket in Selangor were tested for the presence of *V. parahaemolyticus*. All the fish samples were determined positive for *V. parahaemolyticus* using conventional microbiological culture-based method. The *toxR* gene were detected via polymerase chain reaction (PCR) in 165/240 (69%) isolates. The two-virulence factor of *V. parahaemolyticus*, thermostable direct hemolysin (*tdh*) and TDH-related hemolysin (*trh*) was screened via PCR. As such, four isolates were *trh*+and none were *tdh*+. Majority of the isolates presented high resistance to ampicillin (88%), amikacin (64%), and kanamycin (50%). In addition, this study identified 19-imipenem resistant isolates isolated from freshwater and marine fish samples. Further analysis of these 19-imipenem resistant isolates revealed that the resistance toward imipenem was plasmid mediated after plasmid curing assay. The multiple antibiotics resistance index was >0.2 for 70% of the isolates. In summary, the results confirm the presence of *V. parahaemolyticus* in freshwater and marine fish samples in Selangor, Malaysia. To our best knowledge, this is the first report discovering the antibiotic resistant patterns and Carbapenem-resistant isolates of *V. parahaemolyticus* isolated from marine and freshwater fish samples in Selangor.

## Introduction

*Vibrionaceae* family within the class of Gammaproteobacteria comprises of Gram-negative halophilic bacteria, straight or curved rods, ubiquitous and indigenous in aquatic environments (Tison and Kelly, 1984; Tantillo et al., 2004; Sawabe et al., 2013). The *Vibrio* genus consists of 142 species that are marine originated and its taxonomy is continuously been revised due to the discovery of new species (Sawabe et al., 2013). *Vibrio parahaemolyticus* is among the member of this genus that been regarded as important human pathogenic bacteria (Su and Liu, 2007; Iwamoto et al., 2010; Bier et al., 2015; Law et al., 2015). The species is widely distributed in marine and estuarine environments thus leading to gastrointestinal infections upon consumption of raw or undercooked seafood (Kubota et al., 2008; Letchumanan et al., 2014; Lee and Raghunath, 2018). Based on the published data by Centers for Disease Control and Prevention (CDC) in the United States during the year 2016, *V. parahaemolyticus* is considered as a major foodborne bacterium compared to other *Vibrio* species and accounted for nearly 34,664 foodborne cases annually in the United States (Scallan et al., 2011; Huang et al., 2016).

In terms of its pathogenicity, thermostable direct hemolysin (*tdh*) gene, TDH-related hemolysin (*trh*) gene, T3SS systems (T3SS1 and T3SS2) are among the virulence factors own by pathogenic *V. parahaemolyticus* in order to initiate an infection (Letchumanan et al., 2014, 2017). Usually, 99% of clinical *V. parahaemolyticus* isolates are known to be pathogenic because they carry *tdh* genes and/or *trh* genes, whereas majority of the environmental isolates are non-pathogenic (Sudha et al., 2012; Tsai et al., 2013). Nevertheless, around 0–6% of the environmental isolates are identified as pathogenic carrying *tdh* gene and/or *trh* gene (Letchumanan et al., 2014, 2015a).

The aquaculture industry in Malaysia is mainly associated with its economic gains from supplying domestic and foreign demands, and as well as generating a steady income for farmers (Witus and Vun, 2016). Fish is among the popular fishery products that been consumed in daily basis by consumers from Southeast Asian countries (Hajeb et al., 2009). Around 75% of the global fishery production is mainly for human consumptions (Teh, 2012). In Malaysia, the fish consumption has increased since 1970 and now its above 40 kg/capita/year (Teh, 2012). Professed has a healthy food, fish contains a high level of proteins, omega-3 fatty acids (n-3), essential vitamins and minerals that are required by an individual (Aremu and Ekunode, 2008; Hajeb et al., 2009). There are variety of fishes that been consumed by Malaysian in their daily life including the Indian mackerel, Spanish mackerel, black pomfret, silver pomfret, yellowstripe scad, catfish, fringe scale sardine, and tilapia (Osman et al., 2001; Hajeb et al., 2009; Taweel et al., 2013). The expanding and intense aquaculture industry has led to the suppression of immune systems and increases the susceptibility of fish to bacterial infections (Davies et al., 2001; Basti et al., 2006; Harikrishnan et al., 2011).

Intensified fish farming in order to meet consumers demand has prompted the use of antibiotics as treatment regime, prophylaxis and as growth promotion (Vaseeharan et al., 2005). Antibiotics are often been in-cooperated as feed additives or immersion bath in order to treat bacterial infections, promote fast growth of fish, and also prevent the growth of water plants (Abu Bakar et al., 2010). Oxytetracycline, tetracycline, quinolones, sulphonamides, trimethoprim, nalidixic acid, gentamicin, nitrofurazone, and trimethoprim-sulfamethoxazole are among the permitted antibiotics used in the Asian aquaculture industry (Harikrishnan et al., 2011; Manjusha and Sarita, 2011; Rico et al., 2012; Yano et al., 2014). Extensive use of antibiotics in aquaculture has resulted in the increase antibiotic resistance among bacteria including *Vibrio* species (Tendencia and de la Peña, 2001; Jerbi et al., 2011; Heng et al., 2017; Lee and Raghunath, 2018). Direct transmission of resistant bacteria through food to human, and transfer of resistance genes to other bacteria happens, thus causing a possible hazard to human wellbeing (Duran and Marshall, 2005; Guglielmetti et al., 2009; Kim et al., 2013).

Antimicrobial resistance (MDR) has been recognized as an important global threat issue to global public health and food safety (Food and Agriculture Organization [FAO], 2016). In hospitals, many clinical antibiotics are no longer effective to control bacterial infections (Tan et al., 2016). As a result of misuse of antibiotic to control infections during aquaculture production, *V. parahaemolyticus* has been reported to exhibit multidrug resistance, which raised the concern about public health and economic threat of this bacterium (Vaseeharan et al., 2005; Han et al., 2007; Lesley et al., 2011; Manjusha and Sarita, 2011; Noorlis et al., 2011). Carbapenems are always been regarded as the last treatment selection for Gram-positive and Gram-negative infections, and as well as infections caused by multidrug resistant bacteria (Nordmann et al., 2011; Martin et al., 2018). Nevertheless, their use has been compromised causing an increased incidence of carbapenem-resistant bacteria, and widely been discussed among medical practitioners, researchers, and public (Martin et al., 2018). A study by Nordmann and colleagues identified the novel New Delhi metallo-β-lactamase (NDM) encoded by the gene *bla*_NDM-1_ in members of the family *Enterobacteriaceae*. This gene was reported to be not only present largely in *Enterobacteriaceae*, but also in *Vibrionaceae* (Nordmann et al., 2011). Over the years, Carbapenem-resistant *Vibrio* sp. has been detected and isolated from environmental and seafood samples (Walsh et al., 2011; Mandal et al., 2012; Gu et al., 2014; Bier et al., 2015). Recently, in Kolkata, NDM-1 producing *Vibrio fluvialis* strains has been isolated from diarrheal fecal samples from patients (Chowdhury et al., 2016).

The increase in bacterial resistance toward many clinical antibiotics affects many countries healthcare sector and food production sectors (World Health Organization [WHO], 2014). In view of previous reports and the possible severity of infections, continuous investigation on antimicrobial resistance of *V. parahaemolyticus* is needed for epidemiological purpose and guidance in healthcare treatment. For this reason, our study aimed to assess antimicrobial susceptibility profiles of *V. parahaemolyticus* from marine and freshwater fish in Selangor, Malaysia. In addition, we also report the identification and antibiotic resistant characterization of Carbapenem-resistant isolates isolated from marine and freshwater fish samples. To our knowledge, this is the first study examining the antibiotic resistant profiles and Carbapenem-resistant isolates of *V. parahaemolyticus* from both marine and freshwater fish samples in Selangor, Malaysia.

## Materials and Methods

### Sampling

The study focused on two category fish – marine and freshwater fish. A total of 240 fish samples comprising of yellowstripe scad (*Selaroides leptolepis*) (*n* = 48), Indian mackerel (*Rastrelliger kanagurta*) (*n* = 48), black pomfret (*Parastromateus niger*) (*n* = 48), catfish (*Clarias batrachus*) (*n* = 48), and red tilapia (*Oreochromis* spp.) (*n* = 48) were collected from three wet markets and three supermarkets in Selangor (Table 1). From each sampling site, we collected eight fish samples and sampling was done weekly from January 2016 to May 2016. All the samples were kept in sterile sealed bags and transported to the laboratory in an ice box. Samples were analyzed immediately thereafter.

**Table 1:** The frequency of *Vibrio parahaemolyticus* detected in different fish samples from different sampling locations by using PCR assay.

GPS coordinate	Wet market A			Wet market B			Wet market C			Supermarket A			Supermarket B			Supermarket C			Total		
	3°4′ 11.054″N 101°27′ 8.584″E			3°4′ 55.732″N 101°30′ 32.081″E			3°4′ 28.227″N 101°35′ 15.423″E			3°3′ 39.254″N 101°28′ 19.839″E			3°4′ 24.421″N 101°36′ 24.695″E			3°9′ 2.019″N 101°36′ 53.927″E			
Fish sample	*n*	*toxR^+^*	*trh^+^*	*n*	*toxR^+^*	*trh^+^*	*n*	*toxR^+^*	*trh^+^*	*n*	*toxR^+^*	*trh^+^*	*n*	*toxR^+^*	*trh^+^*	*n*	*toxR^+^*	*trh^+^*	*n*	*toxR^+^*	*trh^+^*
Yellowstripe scad	8	8	0	8	6	0	8	6	0	8	4	0	8	5	0	8	6	0	48	35	0
Indian mackerel	8	2	0	8	6	0	8	6	0	8	5	0	8	4	0	8	7	2	48	30	2
Black pomfret	8	4	0	8	5	1	8	7	0	8	3	0	8	6	0	8	6	0	48	31	1
Catfish	8	5	0	8	6	0	8	7	0	8	2	0	8	5	0	8	6	0	48	31	0
Red tilapia	8	7	0	8	5	1	8	7	0	8	5	0	8	7	0	8	7	0	48	38	1
Total	40	26	0	40	28	2	40	33	0	40	19	0	40	27	0	40	32	2	240	165	4

****n*** = number of fish samples purchased from respective location. ***toxR*^+^** = number of positive *Vibrio parahaemolyticus* isolates harboring *toxR* gene. ***trh*^+^** = number of positive *Vibrio parahaemolyticus* isolates harboring *trh* gene.*

### Isolation of *Vibrio* sp. in Fish Samples

Isolation of *Vibrio* sp. was following Standard US Food and Drug Administration (FDA) protocol (Kaysner and DePaola, 2004) and FAO/WHO Risk Assessment of *V. parahaemolyticus* in Seafood (Food and Agriculture Organization/World Health Organization [FAO/WHO], 2011); this method was previously reported by Zarei et al. (2012) and Letchumanan et al. (2015a). 25 g of sample (gut and fish meat) was homogenized with 225 mL of alkaline peptone water (APW) with 2% w/v sodium chloride (NaCl), pH 8.5 for 60 s using a stomacher (BagMixer 400W, Interscience, Saint-Nom-la-Bretèche, France). The homogenate was enriched at 37°C for 18 h. After 18 h of incubation, a loopful of enriched mixture was streaked onto selective media, Thiosulfate Citrate Bile Salts Sucrose (TCBS) agar (HiMedia, India) and incubated at 37°C for 18 h. In each plate, one sucrose non-fermenting colony that has a green or bluish green color measuring about 3–5 mm suggestive of *V. parahaemolyticus* was selected from the TCBS plates. The isolate was purified by re-streaking onto Tryptic Soy Agar (TSA) (HiMedia, India) plates supplemented with 2% w/v sodium chloride (NaCl) (Vivantis, United States). The purified colony were inoculated into TSB semi-solid nutrient agar and stored until further identification.

### DNA Extraction

Bacterial lysate was prepared following established protocol (Suzita et al., 2010; Vengadesh et al., 2012; Letchumanan et al., 2015a,c). The isolates were revived in tryptic soy broth (TSB) (HiMedia) supplemented with 2% w/v sodium chloride NaCl (Vivantis, United States). Overnight suspension was transferred into 1.5 mL of microcentrifuge tube and centrifuged. The supernatant was discarded and 1 mL of sterile ultrapure water was added and vortexed. The suspension was heated at 100°C for 7 min and then cooled on ice immediately into ice for 5 min. Cell debris from the cell lysate were pelleted by centrifugation at 13,000 rpm for 1 min. The supernatant was used as DNA templates for polymerase chain reaction (PCR) assays.

### Identification of *Vibrio parahaemolyticus* Using *toxR*-PCR Assay

Specific primers targeting *toxR* gene with the expected amplicon size of 368 bp were used to identify *V. parahaemolyticus* (Kim et al., 1999; Letchumanan et al., 2015a). The PCR assay was performed in 20 μL reaction mixture containing 2 μL of DNA template, 10 μL of 2× *Taq PLUS* PCR Smart mix 1 (SolGent^TM^, South Korea), 6 μL of ultrapure water and 1 μL of each primer. *toxR*-based PCR amplification was performed using PCR thermocycler (Kyratec, Super Cycler Thermal Cycler, Australia) with the following cycling conditions: initial denaturation at 95°C for 4 min, 35 cycles of 94°C for 1 min, 68°C for 1 min and 72°C for 30 s, and a final elongation at 72°C for 5 min. PCR products was visualized by using 1.5% agarose gel and viewed under UV transilluminator using a Gel Documentation System (ChemiDoc^TM^ XRS, Bio-Rad, United States). The *toxR*-PCR results of a few presumptive *V. parahaemolyticus* isolates and type strain *Vibrio parahaemolyticus* NBRC 12711 were sequenced to confirm the identity of *toxR* gene (Supplementary Table S1). The *Vibrio parahaemolyticus* NBRC 12711 was used as the positive control and *Vibrio vulnificus* NBRC 15645 was used as the negative control.

### Detection of Virulence Gene

Molecular identification of thermostable direct haemolysin (*tdh*) and thermostable-related direct haemolysin (*trh*) was performed using a duplex PCR assay (Bej et al., 1999; Letchumanan et al., 2015c). The PCR assay was done in 20 μL of reaction mixture containing 2 μL of DNA template, 10 μL of 2× *Taq PLUS* PCR Smart mix 1 (SolGent^TM^, South Korea), 4 μL of sterile distilled water and 1 μL of each primer. The PCR amplifications was performed using a Thermocycler (Kyratec, Super Cycler Thermal Cycler, Australia) with the following cycling conditions: initial denaturation at 94°C for 3 min, 30 cycles of 94°C for 1 min, 58°C for 1 min and 72°C for 1 min, and a final elongation at 72°C for 5 min. The PCR products was visualized by using 1.5% agarose gel and viewed under UV transilluminator using a Gel Documentation System (ChemiDoc^TM^ XRS, Bio-Rad, United States). The PCR results of a few presumptive *V. parahaemolyticus* isolates and type strain *Vibrio parahaemolyticus* NBRC 12711 were sequenced to confirm the identity of virulence gene (Supplementary Table S1). *Vibrio parahaemolyticus* NBRC 12711 was used as the positive control and *Vibrio vulnificus* NBRC 15645 was used as the negative control.

### Antibiotic Susceptibility Test

The antibiotic susceptibility of *V. parahaemolyticus* isolates was determined using Kirby-Bauer disk diffusion method (Yano et al., 2014). Fourteen type of antibiotics disks (Oxoid, United Kingdom) was used: amplicon (10 μg), ampicillin/sulbactam (30 μg), amikacin (30 μg), cefotaxime (30 μg), ceftazidime (30 μg), chloramphenicol (30 μg), gentamicin (30 μg), imipenem (10 μg), kanamycin (30 μg), levofloxacin (5 μg), nalidixic acid (30 μg), oxytetracycline (30 μg), sulfamethoxazole/trimethoprim (25 μg), and tetracycline (30 μg). *E. coli* ATCC 25922 with known sensitivity pattern was included as a positive control in each test.

*V. parahaemolyticus* isolates was grown in tryptic soy broth (TSB) (HiMedia, India) 2% w/v sodium chloride (NaCl) (Vivantis, United States) at 37°C for 18 h under constant agitation. The antibiotic disks were dispensed on Mueller Hilton agar (HiMedia, India) supplemented with 2% w/v NaCl (Vivantis, United States) plates with bacterial lawn. After incubation at 37°C for 18 h, the inhibition zone was measured and interpreted based on guidelines of the Clinical and Laboratory Standards Institute (CLSI) M45-A2 (Clinical and Laboratory Standards Institute [CLSI], 2010). The multiple antibiotic resistance (MAR) index was determined based on the ratio of antibiotic resistance exhibited by the isolate to the number of antibiotics to which the isolates were exposed (Krumperman, 1983).

### Plasmid Curing

The 19-imipenem resistant *V. parahaemolyticus* isolate was subjected to plasmid curing assay to determine the antibiotic resistance mediation. The plasmid curing assay was performed using an intercalating agent, ethidium bromide (EB) (Lou et al., 2002; Molina-Aja et al., 2002; Letchumanan et al., 2015b). The isolates were revived in freshly prepared tryptic tryptic soy broth (TSB) supplemented with 0.2 mg/mL EB (Bio Basic, Canada), then incubated at 37°C for 18 h under constant agitation. After treatment with the curing agent, the antibiotic resistance profiles were re-examined and compared with the antibiotic resistance phenotype on non-treated group.

### Genomic and Phylogenetic Analyses

**FIGURE 1:**
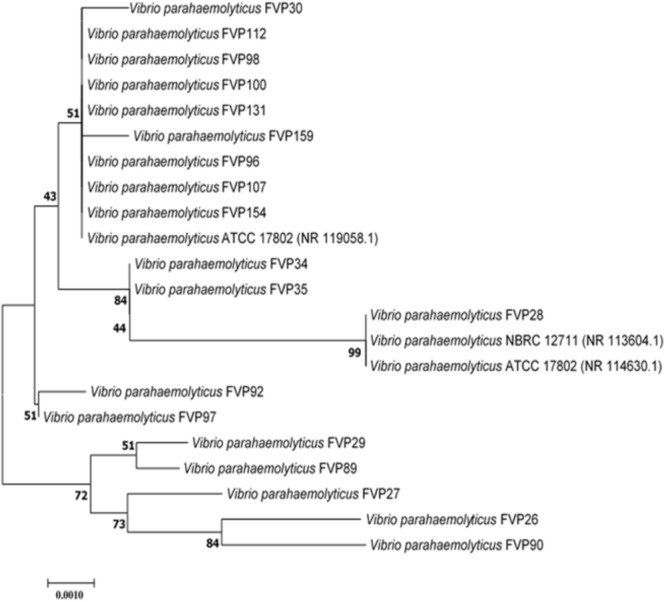
Neighbor-joining phylogenetic tree based on almost complete 16S rRNA sequences showing the relationship between 19-imipenem resistant isolate and representatives of some other related taxa. Numbers at nodes indicate percentages of 1,000 bootstrap re-samplings. Bar: 0.0010 substitutions per site.

Polymerase chain reaction amplification of the 16s rRNA gene for the 19-imipenem resistant *V. parahaemolyticus* was done according to the protocol described by Thomas et al. (2018) with slight modifications. The 16S rRNA gene sequence of each isolate was aligned with representative sequences of related type strains in the genus *V. parahaemolyticus* retrieved from the GenBank/EMBL/DDBJ databases using CLUSTAL-X software (Thompson et al., 1997). The alignment was first verified manually and adjusted, followed by construction of phylogenetic trees with neighbor-joining (Saitou and Nei, 1987; Figure 1) and maximum-likelihood algorithms (Felsenstein, 1981), utilizing the MEGA version 6.0 (Tamura et al., 2013). For neighbor-joining algorithm, the evolutionary distances were computed using the Kimura’s two-parameter model (Kimura, 1980). The calculations of level of sequence similarity were performed by GenBank server^1^. Bootstrap based on 1,000 resampling method of Felsenstein (1985) was used to analyze the stability of the resultant tree topologies.

### Statistical Analysis

Data analysis was performed with SPSS statistical analysis software version 20. Statistical analysis was performed in order to determine whether there was any significant difference in between two types of fish (marine and freshwater fish) and the MAR index of resistant isolates using the independent *t*-test. The significance level was set at *p* ≤ 0.05. One-way analysis of variance (ANOVA) followed by appropriate *post hoc* text (Tukey) was performed to determine the significant differences between the type of fishes and MAR index of resistant isolates. A difference was considered statistically significant when *p* ≤ 0.05.

## Results

### Prevalence of *Vibrio parahaemolyticus* in Fish Samples

The present study isolated *V. parahaemolyticus* from freshwater and marine fish. A total of 240 fish samples comprising of yellowstripe scad (*Selaroides leptolepis*) (*n* = 48), Indian mackerel (*Rastrelliger kanagurta*) (*n* = 48), black pomfret (*Parastromateus niger*) (*n* = 48), catfish (*Clarias batrachus*) (*n* = 48), and red tilapia (*Oreochromis* spp.) (*n* = 48) were collected from three wet market and three supermarkets. Based on the colony morphology on TCBS agar, a total of 240 isolates was picked and purified on TSA agar. The *toxR*-PCR assay exhibited positive amplification of *toxR* gene with 368 bp amplicon band in 69% (165/240) of the presumptive *V. parahaemolyticus* isolates. Based on the sampling location site, 47% (78/165) of the isolates originated from the wet market and 53% (87/165) was from supermarket. A total of 96 (58%) of the isolates were isolated from marine fish samples and 69 (42%) of the isolates were isolated from freshwater fish samples.

### Detection of Thermostable Direct Hemolysin (*tdh*) and *tdh*-Related Hemolysin (*trh*)

A duplex PCR assay was performed to detect the presences of *tdh* and *trh* gene in all isolates (Table 1). None of the 165 *V. parahaemolyticus* isolates yielded *tdh*-positive PCR amplification. Only 4 (2.4%) out of the total 165 *V. parahaemolyticus* showed positive PCR amplification of the *trh* gene. The *trh*-positive *V. parahaemolyticus* isolates was isolated from black pomfret (wet market B) (FVP81), red tilapia (wet market B) (FVP92), and two from Indian mackerel (supermarket C) (FVP47 and FVP49). The presence of *trh*-positive *V. parahaemolyticus* isolates in both types of fish samples indicates possible high risk of foodborne gastroenteritis transmission to humans upon ingestion of the fish.

### Antimicrobial Susceptibilities of *Vibrio parahaemolyticus* Isolates

Most of the tested antibiotics in this study such as tetracycline, folate pathway inhibitors (trimethoprim-sulfamethoxazole), third-generation cephalosporins (cefotaxime and ceftazidime), aminoglycosides (gentamicin and amikacin) and fluoroquinolones (ciprofloxacin and levofloxacin), are among the recommended antibiotics by CDC for the treatment of *Vibrio* sp. infections (Daniels and Shafaie, 2000; Shaw et al., 2014). Table 2 summarizes the percentage of antibiotic resistant profiles of *V. parahaemolyticus* isolated from fish sample. Based on the results, the resistance rate of the 165 *V. parahaemolyticus* isolates in our study was 88% to ampicillin, 64% to amikacin, and 50% to kanamycin. A notable resistance pattern can be observed to the third generation cephalosporins (cefotaxime 52% and ceftazidime 28%). In contrast, high susceptibility rate was seen to chloramphenicol (93%), tetracycline (90%), imipenem (85%), levofloxacin (85%), gentamicin (84%), sulfamethoxazole/trimethoprim (80%), nalidixic acid (78%), oxytetracycline (72%), and ampicillin/sulbactam (70%).

**Table 2:** Percentage of antibiotic susceptible, intermediate, and resistant of *V. parahaemolyticus* isolated from various fish samples.

Antibiotics	Susceptible (S)		Intermediate (I)		Resistant (R)		Total no. of isolates
	No. of isolates	%^a^	No. of isolates	%	No. of isolates	%
**Penicillins & β-Lactam/β-Lactamase inhibitor combinations**							
Ampicillin (AMP10)	12	7	8	5	145	88	165
Ampicillin/Sulbactam (SAM30)	115	70	14	8	36	22	165
**Phenicols**							
Chloramphenicol (C30)	154	93	7	4	4	2	165
**Tetracyclines**							
Oxytetracycline (OT30)	118	72	18	11	29	18	165
Tetracycline (TE30)	149	90	3	2	13	8	165
**Folate Pathway Inhibitor**							
Sulphamethox/Trimethoprim (SXT25)	132	80	25	15	8	5	165
**Cephems**							
Cefotaxime (CTX30)	46	28	33	20	86	52	165
Ceftazidime (CAZ30)	80	48	39	24	46	28	165
**Carbapenems**							
Imipenem (IPM10)	140	85	6	4	19	12	165
**Aminoglycosides**							
Amikacin (AK30)	23	14	36	22	106	64	165
Kanamycin (K30)	21	13	62	38	82	50	165
Gentamicin (CN30)	138	84	25	15	2	1	165
**Quinolones**							
Nalidixic acid (NA30)	128	78	23	14	14	8	165
Levofloxacin (LEV5)	140	85	24	15	1	1	165

*^*a*^% = percentage (number of isolates/total number of isolates tested).*

Interestingly, 19 isolates (12%) from this study exhibited resistance to imipenem, an antibiotic in Carbapenem class. The detection of imipenem resistant isolates is of concern as Carbapenems are among the beta-lactams that is the last line antibiotic used for bacterial infection treatment (Meletis, 2016). These 19 isolates had an MAR index of 0.14 to 0.50, resistant to more than two different type of antibiotics tested. Majority of the imipenem resistant isolates were isolate from freshwater fish sample (15/19) and the remaining 4 isolates were isolated from marine fish samples.

**FIGURE 2:**
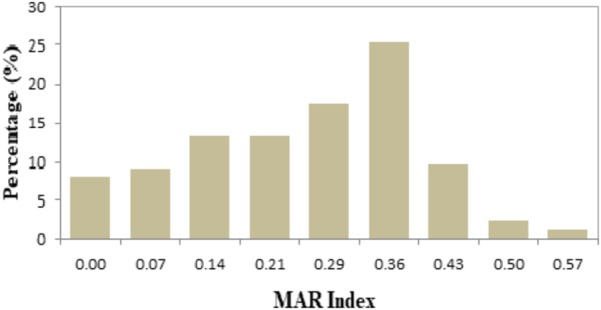
Percentage occurrence of MAR index of *Vibrio parahaemolyticus* isolates from all fish samples of different sampling locations. The isolates exhibited MAR index from 0.00 to 0.57, representing that the *V. parahaemolyticus* isolates are resistant to 0–8 types of antibiotics tested.

In this study, the values of MAR index ranged from 0.00 to 0.57 (Table 3). Forty-two different resistance patterns had a significant MAR index more than 0.2. Two of the isolates (FVP24 – yellowstripe scad, marine fish and FVP67 – red tilapia, freshwater fish) has the highest MAR index of 0.57, resistant to 8/14 antibiotics tested. Further analysis was performed by comparing the MAR index between source of sample (marine and freshwater) and MAR index. The mean MAR index of marine fish sample was 0.26 where else, freshwater fish sample was 0.25. The results showed that there was no significant difference between source of fish sample and MAR index. According to the one-way ANOVA analysis, there was no significant difference between the fish types on the MAR index of *V. parahaemolyticus* isolates. The results suggest that isolates from the fish samples may have similar level of antibiotic exposure, regardless there are marine or freshwater originated. As shown in Figure 2, 8% of *V. parahaemolyticus* isolates (13 isolates) did not exhibited MAR as they were susceptible to all of the antibiotics tested.

**Table 3:** Antibiogram and multiple antimicrobial resistance (MAR) indices of *V. parahaemolyticus* isolates.

No.	Antibiotic resistant pattern	MAR index	Number of *V. parahaemolyticus* isolates
1	AMP	0.07	15
2	AMP/AK	0.14	8
3	AMP/CTX	0.14	5
4	AMP/IPM	0.14	1
5	AMP/K	0.14	1
6	AMP/OT	0.14	2
7	AMP/SAM	0.14	2
8	AK/CTX	0.14	1
9	OT/TE	0.14	1
10	AK/SXT	0.14	1
11	AMP/AK/K	0.21	6
12	AMP/AK/CTX	0.21	5
13	AMP/CTX/IPM	0.21	2
14	AMP/IPM/OT	0.21	1
15	AMP/OT/TE	0.21	1
16	AMP/AK/SAM	0.21	1
17	AMP/IPM/SAM	0.21	5
18	AMP/NA/SAM	0.21	1
19	AMP/C/OT/TE	0.29	1
20	AMP/AK/CN/K	0.29	1
21	AMP/AK/CTX/K	0.29	16
22	AMP/CTX/IPM/OT	0.29	1
23	AMP/AK/OT/TE	0.29	2
24	AMP/AK/K/SAM	0.29	2
25	AMP/AK/IPM/SAM	0.29	1
26	AMP/IPM/OT/SAM	0.29	1
27	AMP/OT/NA/SAM	0.29	1
28	AK/CTX/K/OT	0.29	1
29	AK/K/OT/TE	0.29	1
30	AK/CTX/K/TE	0.29	1
31	AMP/C/CTX/OT/TE	0.36	1
32	AMP/AK/CAZ/CTX/K	0.36	33
33	AMP/AK/CTX/OT/SXT	0.36	1
34	AMP/AK/K/OT/TE	0.36	1
35	AMP/NA/OT/TE/SXT	0.36	1
36	AMP/AK/K/NA/SAM	0.36	1
37	AMP/AK/K/IPM/SAM	0.36	1
38	AMP/IPM/OT/NA/SAM	0.36	1
39	AMP/CTX/OT/NA/SAM	0.36	1
40	AMP/AK/NA/SAM/SXT	0.36	1
41	AMP/AK/CTX/K/IPM/OT	0.43	2
42	AMP/AK/CAZ/CTX/K/SAM	0.43	10
43	AMP/AK/K/IPM/NA/SAM	0.43	1
44	AMP/AK/CTX/OT/NA/SAM	0.43	1
45	AMP/AK/IMP/NA/OT/SAM	0.43	1
46	AMP/C/NA/OT/SXT/TE	0.43	1
47	AMP/AK/C/CTX/NA/OT/SXT	0.5	1
48	AMP/AK/CAZ/CTZ/K/LEV/SAM	0.5	1
49	AMP/AK/CTX/IPM/K/OT/SAM	0.5	1
50	AK/CAZ/CN/CTX/OT/SXT/TE	0.5	1
51	AMP/AK/CAZ/CTX/K/NA/OT/SAM	0.57	1
52	AMP/AK/K/NA/OT/SAM/SXT/TE	0.57	1

*AMP, amplicon; OT, oxytetracycline; NA, nalidixic acid; C, chloramphenicol; CTX, cefotaxime; SXT, sulfamethoxazole/trimethoprim; IMP, imipenem; AK, amikacin; SAM, ampicillin/sulbactam; LEV, levofloxacin; CAZ, ceftazidime; K, kanamycin; CN, gentamicin; TE, tetracycline.*

The antibiotic resistance patterns between freshwater and marine fish samples did not exhibit any significant profiles. Based on the analysis, both freshwater and marine fish samples were exposed to antibiotics and phenotypic assay showed a similar resistant profile to 0 to 8 types of antibiotics tested. For each fish sample, the mean MAR indices for *V. parahaemolyticus* isolates was 0.26 for yellowstripe scad, Indian mackerel was 0.24, black pomfret was 0.29, catfish was 0.25, and red tilapia was 0.24.

### Plasmid Curing

Plasmid curing may server as an effective assay to determine the antibiotic resistance mediation of bacteria. This assay enables to eliminate desired bacterial plasmid and subsequently reassess the antibiotic resistance phenotype by antibiotic disk diffusion method. Table 4 summarizes the antibiotic resistance profile of the 19-imipenem resistant isolates before and after plasmid curing assay. All the phenotypically seen imipenem resistant isolates became susceptible to imipenem after curing assay, suggesting the resistance was plasmid mediated. All the isolates were still resistant to ampicillin and oxytetracycline, suggesting a possible chromosomal mediated resistance. Hence, the antibiotic resistance seen in 19-imipenem isolates are both plasmid and chromosomally mediated.

**Table 4:** List of 19-imipenem resistant *Vibrio parahaemolyticus* isolates.

				Before plasmid curing	After plasmid curing
No	Isolate	Fish sample	Source	Antibiotic resistance pattern	Antibiotic resistance pattern
1	FVP26	Red tilapia	Freshwater	AMP/IMP/SAM	AMP
2	FVP27	Red tilapia	Freshwater	AMP/IMP/SAM	AMP
3	FVP28	Red tilapia	Freshwater	AMP/IMP/SAM	AMP
4	FVP29	Red tilapia	Freshwater	AMP/IMP/SAM	AMP
5	FVP30	Red tilapia	Freshwater	AMP/IMP/SAM	AMP
6	FVP34	Catfish	Freshwater	AMP/AK/IMP/SAM	AMP
7	FVP35	Catfish	Freshwater	AMP/IMP/SAM	AMP
8	FVP89	Yellowstripe scad	Marine	AMP/AK/IMP/K/SAM	AMP
9	FVP90	Yellowstripe scad	Marine	AMP/IMP	AMP
10	FVP92	Red tilapia	Freshwater	AMP/AK/CTX/IMP/K/OT	AMP/OT
11	FVP96	Red tilapia	Freshwater	AMP/IMP/OT/NA/SAM	AMP/OT
12	FVP97	Red tilapia	Freshwater	AMP/AK/IMP/K/NA/SAM	AMP
13	FVP98	Catfish	Freshwater	AMP/IMP/OT	AMP/OT
14	FVP100	Catfish	Freshwater	AMP/IMP/OT/SAM	AMP/OT
15	FVP107	Catfish	Freshwater	AMP/AK/CTX/IMP/K/OT	AMP/OT
16	FVP112	Red tilapia	Freshwater	AMP/AK/CTX/IMP/K/OT/SAM	AMP/OT
17	FVP131	Yellowstripe scad	Marine	AMP/CTX/IMP/OT	AMP/OT
18	FVP154	Indian mackerel	Marine	AMP/CTX/IMP	AMP
19	FVP159	Red tilapia	Freshwater	AMP/CTX/IMP	AMP

*AMP, amplicon; OT, oxytetracycline; NA, nalidixic acid; C, chloramphenicol; CTX, cefotaxime; SXT, sulfamethoxazole/trimethoprim; IMP, imipenem; AK, amikacin; SAM, ampicillin/sulbactam; LEV, levofloxacin; CAZ, ceftazidime; K, kanamycin; CN, gentamicin; TE, tetracycline.*

### Genomic and Phylogenetic Analyses

The nearly complete 16S rRNA gene sequence was determined for all the 19-imipenem resistant *V. parahaemolyticus* isolates and manual alignment of these sequences was performed with the corresponding partial 16S rRNA gene sequences of the type strains of *V. parahaemolyticus* retrieved from GenBank/EMBL/DDBJ databases. Phylogenetic tree was constructed based on the 16S rRNA gene sequences to determine the phylogenetic position of the 19-imipenem resistant isolates (Figure 1). Phylogenetic analysis exhibited that closely related strains include *Vibrio parahaemolyticus* ATCC 17802 (NR 119058.1), *Vibrio parahaemolyticus* NBRC 12711 (NR 113604.1) and *Vibrio parahaemolyticus* ATCC 17802 (NR 114630.1), as the 19-imipenem resistant isolates form distinct five clades. The isolates within the same clade are closely related.

## Discussion

The occurrence of pathogenic strains of *V. parahaemolyticus* in fish samples we studied does raise concern as this organism is known to cause foodborne gastroenteritis resulted from ingesting of uncooked or undercooked seafood (Ma et al., 2014; Romalde et al., 2014). However, while the microbiological culture-based method found all fish samples to be contaminated *Vibrio* sp., only 69% (165/240) of there were confirmed to be *V. parahaemolyticus* based on *toxR* PCR assay; and only 2.4% (4/165) of these were pathogenic strains (*trh*-positive) (Table 1). Our results came to an agreement with other researchers on the fact that the identity of *V. parahaemolyticus* could not be fully confirmed by conventional microbiological culture-based method (Kim et al., 1999; Zulkifli et al., 2009; Fabbro et al., 2010; Ottaviani et al., 2013). Affirming with previous research, we found that *toxR* PCR assay was specific and reliable technique for the identification of both pathogenic and non-pathogenic *V. parahaemolyticus* (Kim et al., 1999; Dileep et al., 2003; Zulkifli et al., 2009). This reliable and specific *toxR*-PCR assay has resulted in many promising *V. parahaemolyticus* identifications studies (Deepanjali et al., 2005; Das et al., 2009; Vimila et al., 2010; Elamparithi and Ramanathan, 2011; Noorlis et al., 2011; Paydar et al., 2013). The remaining 75 isolates had the morphology of *V. parahaemolyticus* in TCBS agar, however, the *toxR* gene was not present in these isolates. This result demonstrates the detection of *V. parahaemolyticus* thru *toxR* PCR assay is highly sensitive, specific and accurate compared to microbiological culture-based technique (Mandal et al., 2011).

The *tdh* and *trh* genes are considered major virulence factors in *V. parahaemolyticus*, so in many clinically isolated strains of *V. parahaemolyticus* have hemolytic activity that is produced by these two genes (Ceccarelli et al., 2013; Raghunath, 2015). Our study reported the isolation of *trh*-positive isolates of *V. parahaemolyticus* at a very low prevalence rate, and none of the isolates have *tdh*-position genes. Our results follow the trends of worldwide dispersed studies that have reported low number of virulent *V. parahaemolyticus* strains from environmental sources (Fuenzalida et al., 2006; Nordstrom et al., 2007; Thongjun et al., 2013). Many studies have reported low prevalence rate (less than 5%) of environmental and food source have pathogenic *V. parahaemolyticus* isolates carrying *tdh* and/or *trh* genes (Parveen et al., 2008; Zulkifli et al., 2009; Tsai et al., 2013). In addition, it is strongly suggested that putative pathogenic environmental *V. parahaemolyticus* isolates may be less virulent than the clinical *V. parahaemolyticus* isolates (Vongxay et al., 2008; Tsai et al., 2013). The presences of *tdh*+ and/or *trh*+ *V. parahaemolyticus* in the marine and freshwater fish samples in Selangor is of concern due to several factors. Firstly, the fact that these pathogenic isolates could potentially cause gastroenteritis (Jun et al., 2012). Secondly, pathogenic *V. parahaemolyticus* not only contaminate seafood and transmit pathogenesis, but it also causes huge economic loss in the aquaculture sector (Fuenzalida et al., 2006; Thongjun et al., 2013). Hence, the study results need the importance for continuous monitoring of seafood for pathogen contamination.

Our antibiotic susceptibility test placed ampicillin at the top of the *V. parahaemolyticus* resistance scope (88%). This finding signifying that ampicillin may longer be an effective antibiotic to treat *Vibrio* sp. infections. In fact, *V. parahaemolyticus* resistance to ampicillin is well reported in many literatures (Joseph et al., 1978; Lesmana et al., 2001; Zulkifli et al., 2009; Melo et al., 2011; Oh et al., 2011; Al-Othrubi et al., 2014). Interestingly, ampicillin resistance was reported 100% in study by Devi et al. (2009) and Ottaviani et al. (2013). The chromosomally encoded β-lactamase is the cause for *V. parahaemolyticus* resistance to ampicillin and other penicillin (Devi et al., 2009). In addition, more that 70% of the *V. parahaemolyticus* isolates in this study remained susceptible to tetracycline, levofloxacin, gentamicin, sulfamethoxazole/trimethoprim, chloramphenicol, imipenem, nalidixic acid, oxytetracycline, and ampicillin/sulbactam. Our findings are in line with previous studies that reported susceptibility of *V. parahaemolyticus* against chloramphenicol, tetracyclines, trimethoprim-sulfamethoxazole, nalidixic acid, and imipenem (Ottaviani et al., 2001; Devi et al., 2009; Melo et al., 2011; Al-Othrubi et al., 2014; Shaw et al., 2014). The MAR index values ranged from 0 to 0.57.

Forty-two different resistance patterns had a significant MAR value >0.2. Collectively, there were expressed by 70% of the *V. parahaemolyticus* isolates and resistant to 3 to 8 types of antibiotics tested. MAR index >0.2 are exposed to several antibiotics or isolated from contaminated sources as such dairy cattle, aquaculture, and agriculture farms. Where else, isolates with lesser than 0.2 MAR indices are lessen prone to antibiotic exposure (Noorlis et al., 2011; Subramani and Vignesh, 2012). In this study there was no significant difference been observed among the source of sample and MAR index. This result demonstrates that the isolates isolated from marine and freshwater samples are exposure of antibiotics. Our results came to an agreement with many studies that reported high percentage of *V. parahaemolyticus* isolated from seafood are resistant to more than one antibiotic tested (Zulkifli et al., 2009; Lesley et al., 2011; Manjusha and Sarita, 2011; Noorlis et al., 2011).

Imipenem, a member of Carbapenem class is an effective antibiotic used in the treatment of Gram-positive and Gram-negative infections (Papp-Wallace et al., 2011). Interestingly, in this study we detected 19-imipenem resistant *V. parahaemolyticus* strains isolated from marine and freshwater fish samples. Ever since the first detection case of carbapenemase producing Carbapenem-Resistant Enterobacteriaceae (Cp-Cre) in the United States, Cp-Cre have rapidly spread with more reported cases in another 50 states (Centers for Disease Control and Prevention [CDC], 2018). In fact, now carbapenem resistance is no longer associated with Enterobacteriaceae but also associated with other bacteria. As such, the resistance of *Vibrio* sp. to carbapenem has been reported by Bier et al. (2015) in Germany coastal line, Gu et al. (2014) in Southwest China, and Walsh et al. (2011) in India. Thus, our results agree with other findings and demonstrates the misuse of carbapenem that may cause a negative impact on the clinical treatment of *Vibrio* infections in future. Hence, a non-antibiotic approach is required in order to manage the occurrence of antibiotic resistance among *Vibrio* sp. in the environments (Tan et al., 2014; Letchumanan et al., 2016; Tan et al., 2016).

Further analysis on the 19-imipenem resistant isolates by plasmid curing assay exhibited interesting findings. The antibiotic resistance phenotype of these 19 isolates have been altered after plasmid curing. All the 19 isolate’s phenotypically seen resistance to imipenem has changed to susceptible, suggesting the resistance was plasmid mediated. All the isolates were still resistant to ampicillin, suggesting the resistance was chromosomal mediated. In addition, the isolate FVP92, FVP96, FVP98, FVP100, FVP107, FVP112, FVP131, FVP154, FVP159 (Table 3) remained resistant to oxytetracycline even after plasmid curing and its chromosomally mediated. It is usual to find oxytetracycline resistant isolates from aquaculture products because this antibiotic is among the permitted antimicrobial used in the seafood production. In summary, plasmids are transferable between different bacteria and the presence of antibiotic resistant genes in the bacterial plasmid have facilitated the fast spreading of antibiotic resistance among bacteria (Wilson and Salyers, 2003; Stepanauskas et al., 2006; Manjusha and Sarita, 2011). Hence, the acquisition of imipenem resistance by the 19 isolates are possibly due to horizontal gene transfer from other environmental bacteria.

The results from phylogenetic and genomic analyses indicated that the 19-imipenem resistant isolates are closely related forming five clades (Figure 1). The isolates are closely related to each another within the same clade. Isolate FVP28 was closely related to *Vibrio parahaemolyticus* NBRC 12711 and *Vibrio parahaemolyticus* ATCC 17802 at 99% bootstrap value, indicating the high confident level of the association. The isolate FVP28 and both type strains are isolated from food source. The FVP28 was isolated from freshwater red tilapia where else, *Vibrio parahaemolyticus* Nbrd 12711 and *Vibrio parahaemolyticus* ATCC 17802 was originally isolated from shirasu food poisoning case in Japan. This result exhibits a close relationship between these strains isolated from different types of seafood. In addition, there was another clade with nine isolates (FVP30, FVP112, FVP98, FVP100, FVP131, FVP159, FVP96, FVP107, and FVP154) that were closely related to *Vibrio parahaemolyticus* ATCC 17802 at 51% bootstrap value. 7/9 of the isolates (FVP30, FVP112, FVP98, FVP100, FVP159, FVP96, and FVP107) was isolated from freshwater fish sample. Majority of the isolates within this clade were resistant to oxytetracycline, an antibiotic that is permitted in Asian aquaculture industry (Yano et al., 2014). In summary, phylogenetic tree analysis revealed that there was no distinctive grouping based on the antibiogram of each isolate, however, the16S rRNA sequencing had a high discriminating power to group the isolates into different clades (Hoffmann et al., 2010).

The global increase of antibiotic resistant bacteria is of great public health concern and warrants a continuous monitoring (Xie et al., 2017). In the case of *V. parahaemolyticus*, the situation is aggravated due to excessive use of antimicrobial agents in aquaculture to protect infectious diseases and huge production loses (Xu et al., 2016). In addition, antimicrobial resistance is likely caused by exposure to antibiotics via agriculture runoff or wastewater treatment plants, and thru mobile genetic elements or horizontal gene transfers among bacteria (Stepanauskas et al., 2006; Kümmerer, 2009; Al-Othrubi et al., 2014; Xu et al., 2016). Recently, the Food and Agriculture Organization (FAO) have drawn action plans to increase awareness and promote prudent use of antimicrobials (Food and Agriculture Organization [FAO], 2015).

## Conclusion

Our study confirms the presences of *V. parahaemolyticus* in freshwater and marine fish samples in Selangor by having use highly accurate detection and identification method (the combination of microbiological culture-based method and PCR). To our best knowledge, this study represents the first evidence of Carbapenem resistant isolate and as well as antibiotic resistance patterns of *V. parahaemolyticus* isolated from freshwater and marine fish samples. The detection of *tdh* and *trh* genes provides better understanding regarding the distribution of pathogenic *V. parahaemolyticus* strains in fish samples. Despite the fact that majority most of the environmental *V. parahaemolyticus* isolates are non-pathogenic, consumer should still be aware and ensure that fish is cooked properly before consumption. Adequate cooking of fish before consumption is the main safety measure to prevent foodborne disease caused by *V. parahaemolyticus* associated with fish (Zulkifli et al., 2009). Furthermore, several important measures including good hygiene practices while handling the fish and the cleanliness of the handlers and display area are very crucial in order to prevent cross-contamination in wet market and supermarket. In conclusion, the information presented serves as a baseline on future microbiological risk assessment of *V. parahaemolyticus* associated with fish consumption in Selangor, Malaysia.

## Author Contributions

L-HL, VL, and JL conducted the experiments and data analysis, and wrote the manuscript. N-SAM and SW provided vital insight, technical support, guidance, and proofreading for the project. L-HL founded the project.

## Conflict of Interest Statement

The authors declare that the research was conducted in the absence of any commercial or financial relationships that could be construed as a potential conflict of interest.

## Footnotes

1 https://blast.ncbi.nlm.nih.gov
