# Supplementary material for: Discovery on Antibiotic Resistance Patterns of Vibrio parahaemolyticus in Selangor Reveals Carbapenemase Producing Vibrio parahaemolyticus in Marine and Freshwater Fish
Source: Front Microbiol. 2018 Oct 25;9:2513. doi: 10.3389/fmicb.2018.02513 (PMC6209626; doi:10.3389/fmicb.2018.02513)
Supplement: Supplementary file 1 [file Data_Sheet_1.PDF]

Table S1 The sequencing results of selected *V. parahaemolyticus* isolates and NCBI blast similarity

Isolate name	Targeted Gene	Consensus Sequence	Sequence Length	Blast Similarity Results	
				Hit Strain	Similarity Precentage
FVP26	*toxR*	ACGCAATCGTTGAACCAGAAGCGCCAGTAGTACCT<br>GAAAAAGCACCTGTGGCTTCTGCTGTGAATCCTTG<br>GATTCCACGCGTTATTTTATTTTTGGCACTATTACTA<br>CCGATTTGCGTACTGCTGTTTACAAACCCTGCGGA<br>ATCTCAGTTCCGTCAGATTGGTGAGTATCAGAACG<br>TACCAGTGATGACACCTGTAAATCACCCGCAAATC<br>AACAACTGGTTGCCTTCTATTGAGCAGTGCATTGA<br>ACGCTACGTTAAGCACCATGCAGAAGACTCGTTAC<br>CAGTGGAAGTAATTGCCACTGGCGGACAAAATAAC<br>CAGCTGATTTTGAACTACATTCATGACAGCAA	349	*Vibrio parahaemolyticus* strain ATCC 17802, (CP014046.2)	100%
FVP35	*toxR*	TTCTGCTGTGAATCCTTGGATTCCACGCGTTATTTT<br>ATTTTTGGCACTATTACTACCGATTTGCGTACTGCT<br>GTTTACAAACCCTGCGGAATCTCAGTTCCGTCAGA<br>TTGGTGAGTATCAGAACGTACCAGTGATGACACCT<br>GTAAATCACCCGCAAATCAACAACTGGCTGCCTTC<br>TATTGAGCAGTGCATTGAACGCTACGTTAAGCACC<br>ATGCAGAAGACTCGTTACCAGTGGAAGTGATTGCC<br>ACTGGTGGACAAAATAACCAGCTGATTTTGAACTA<br>CATTCATGACAGCAACCA	300	*Vibrio parahaemolyticus* strain VP4 ToxR regulatory protein (toxR) gene, (MF983557.1)	99%
FVP47	*trh*	TACTTCGCGATGATCTGCATCATACCTTTTCCTTCT<br>CCAGGGTCGGCTGAACTGTTATTTGTTGTTAGAAAT<br>ACAACAATCAAAACTGAATCCCCGGTTAAGGCAAT<br>TGTGGAGGACTATTGGACAAACCGAAACATAAAAA<br>GAAAACCATACAAAGATGTATACGGTCAATCGGTT<br>TTCACAACAGCAGGTTCAAAGTGGTTAAGCGCCTA<br>TATGACAGTAAACATCAATGGTCATAACTATACGA<br>TGGCAGCTCTTTCTGGTTATAAAGATGGTATTTCTA<br>CGGTCTTCACAAAATCAGAGAAAACAAGCCTAAA<br>GCAAGACTATTCCTCGGTAAAATATTTTGTTGATGA<br>CAACGAAGAATCAATACCAAGTGTAACTTATTTAG<br>ATGAAACATCAGAATACTTTGTTACTGTCGAGGCA<br>TATGAGAGCGGAAATGGGCATAGTTTTGTTATGA	457	*Vibrio parahaemolyticus* strain ATCC 17802 thermostable direct hemolysin-related hemolysin (trh) gene, (GU971654.1)	98%
